# Supplementary material for: A single-cell atlas of the testicular interstitium defines Leydig progenitor networks sustaining Leydig cell homeostasis across the lifespan
Source: eLife. 2025 Dec 23;14:e100396. doi: 10.7554/eLife.100396 (PMC12826670; doi:10.7554/eLife.100396)
Supplement: Supplementary file 1. [file elife-100396-supp1.docx]

**Supplementary File 1.** **shRNAs and primers**

| **shRNA name** | **Sequence: 5’-3’** |
| --- | --- |
| mouse Sox4-shRNA-1 | CCGG-TGAAGCGCGTCTACCTGTTTG-CTCGAG-CAAACAGGTAGACGCGCTTCA-TTTTTT |
| mouse Sox4-shRNA-2 | CCGG-CGAGATGATCTCGGGAGATTG-CTCGAG-CAATCTCCCGAGATCATCTCG-TTTTTT |
| mouse Sox4-shRNA-3 | CCGG-GCGAGATGATCTCGGGAGATT-CTCGAG-AATCTCCCGAGATCATCTCGC-TTTTTT |
| Sox4-shControl | CCGG-GATTCTCCGAACGTGTCACGTCTCGAG-ACGTGACACGTTCGGAGAATC-TTTTTT |
| mouse Cd34-shRNA-1 | CCGG-CTTGGGCACCACTGGTTATTT-CTCGAG-AAATAACCAGTGGTGCCCAAG-TTTTTT |
| mouse Cd34-shRNA-2 | CCGG-GACCCTTATTACACGGAGAAT-CTCGAG-ATTCTCCGTGTAATAAGGGTC-TTTTTT |
| mouse Cd34-shRNA-3 | CCGG-CGAGTGCCATTAAGGGAGAAA-CTCGAG-TTTCTCCCTTAATGGCACTCG-TTTTTT |
| Cd34-shControl | CCGG-GATTCTCCGAACGTGTCACGT-CTCGAG ACGTGACACGTTCGGAGAATC-TTTTTT |
| **Primer name** | **Sequence: 5’-3’** |
| Sox4-F | CGGCTGCATCGTTCTCTCC |
| Sox4-R | GGTAGACGTGCTTCACTTTCTTG |
| Cd34-F | GGTAGCTCTCTGCCTGATGAG |
| Cd34-R | TGGTAGGAACTGATGGGGATATT |
| Lhcgr-F | CGCCCGACTATCTCTCACCTA |
| Lhcgr-R | GACAGATTGAGGAGGTTGTCAAA |
| Hsd3b1-F | TGGACAAAGTATTCCGACCAGA |
| Hsd3b1-R | GGCACACTTGCTTGAACACAG |
| Star-F | ATGTTCCTCGCTACGTTCAAG |
| Star-R | CCCAGTGCTCTCCAGTTGAG |
| Cyp11a1-F | AGGTCCTTCAATGAGATCCCTT |
| Cyp11a1-R | TCCCTGTAAATGGGGCCATAC |
| Cyp17a1-F | GCCCAAGTCAAAGACACCTAAT |
| Cyp17a1-R | GTACCCAGGCGAAGAGAATAGA |
