# Supplementary material for: A single-cell atlas of the testicular interstitium defines Leydig progenitor networks sustaining Leydig cell homeostasis across the lifespan
Source: eLife. 2025 Dec 23;14:e100396. doi: 10.7554/eLife.100396 (PMC12826670; doi:10.7554/eLife.100396)
Supplement: Supplementary file 2. [file elife-100396-supp2.docx]

**Supplementary File 2. Primary and secondary antibodies**

| **Primary antibodies** | | |
| --- | --- | --- |
| anti-CD34 | Abcam | Cat# ab81289 |
| anti-Adgre1(F4-80)-Rabbit | Cell Signaling Technology | Cat# 30325T |
| 3β-HSD (A-1) | Santa Cruz Biotechnology | Cat# sc-515120 |
| anti-Sycp3 | Santa Cruz Biotechnology | Cat# sc-74569 |
| anti-Lhcgr | Santa Cruz Biotechnology | Cat# sc-393592 |
| anti-Sox4 | Abcam | Cat# ab243739 |
| anti-mouse CD34-FITC | BD | Cat# 553733 |
| anti-mouse CD36-APC | BioLegend | Cat# 102612 |
| anti-mouse CD34-PE | BioLegend | Cat# 128610 |
| Anti-Histone H3 (acetyl K27) | abcam | Cat# ab4729 |
| Anti-Cyp51 | abcam | Cat# ab210792 |
| Anti-Col1a1 | abcam | Cat# ab21286 |
| **Secondary antibodies** | | |
| Goat Anti-Rabbit AF594 | Thermo Fisher Scientific | Cat# A11037 |
| Goat Anti-Rabbit AF488 | Thermo Fisher Scientific | Cat# A11034 |
| Goat Anti-Mouse AF594 | Thermo Fisher Scientific | Cat# A11032 |
| Goat Anti-Mouse AF488 | Thermo Fisher Scientific | Cat# A11029 |
