# Supplementary material for: A single-cell atlas of the testicular interstitium defines Leydig progenitor networks sustaining Leydig cell homeostasis across the lifespan
Source: eLife. 2025 Dec 23;14:e100396. doi: 10.7554/eLife.100396 (PMC12826670; doi:10.7554/eLife.100396)
Supplement: Supplementary file 3. [file elife-100396-supp3.docx]

**Supplementary File 3. Chemicals, Peptides, and Recombinant Proteins**

| DPBS | Thermo Fisher Scientific | Cat# 14190-144 |
| --- | --- | --- |
| Fetal Bovine Serum | Thermo Fisher Scientific | Cat# 9002-93-1 |
| Type IV Collagenase | Thermo Fisher Scientific | Cat# 17104019 |
| Hoechst 33342 | Thermo Fisher Scientific | Cat# H21492 |
| DMEM/F-12 (phenol red-free) | Thermo Fisher Scientific | Cat# 21041025 |
| Penicillin-Streptomycin | Thermo Fisher Scientific | Cat# 15140-122 |
| KnockOut Serum Replacement | Thermo Fisher Scientific | Cat# 10828-028 |
| NEAA | Thermo Fisher Scientific | Cat# 11140050 |
| Insulin-Transferrin-Selenium | Thermo Fisher Scientific | Cat# 41400045 |
| GlutaMAX | Thermo Fisher Scientific | Cat# 35050061 |
| N2 | Thermo Fisher Scientific | Cat# A1370701 |
| B27 | Thermo Fisher Scientific | Cat# A1486701 |
| β-mercaptoethanol | Merck | Cat# ES-007-E |
| BSA | AMRESCO | Cat# 0332-100G |
| Gelatin | AMRESCO | Cat# 9764 |
| Triton X-100 | AMRESCO | Cat# 9002-93-1 |
| 0.25%Trypsin | Corning | Cat# 25-053-CI |
| Recombinant mouse PDGF-BB | PeproTech | Cat# 315-18-10 |
| Recombinant mouse LIF | PeproTech | Cat# 250-02-25 |
| Recombinant mouse bFGF | PeproTech | Cat# 450-33-50 |
| Recombinant mouse TGF-β | PeproTech | Cat# 100-21-2 |
| Recombinant mouse IGF1 | PeproTech | Cat# 100-11-100 |
| Recombinant murine EGF | PeproTech | Cat# 315-09 |
| Luteinizing hormone (LH) | PeproTech | Cat# 12-4208 |
| Oncostatin M | R&D | Cat# 28-11178-2 |
| 3,3′,5′-Triiodo-L-thyronine (TH) | Sigma | Cat# T2877 |
| Dexamethasone | Sigma | Cat# D4902 |
| 4% paraformaldehyde, PFA | Dingguo, China | Cat# AR-0211 |
| Ethylene dimethanesulfonate | Shaoyuan, China | Cat# EN300-137131 |
| Chicken Embryo Extract | absin | Cat# abs80002 |
| Gentian Violet solution | Sigma-Aldrich | Cat# 49144 |
| Goat Serum for Blocking | Beijing ZhanShanJinQiao | Cat# ZLI-9022 |
| Hematoxylin | Beijing ZhanShanJinQiao | Cat# ZLI-9610 |
| Eosin | Beijing ZhanShanJinQiao | Cat# ZLI-9612 |
| human Chorionic gonadotropin (hCG) | Sigma-Aldrich | Cat# 1297001 |
