# Supplementary material for: A single-cell atlas of the testicular interstitium defines Leydig progenitor networks sustaining Leydig cell homeostasis across the lifespan
Source: eLife. 2025 Dec 23;14:e100396. doi: 10.7554/eLife.100396 (PMC12826670; doi:10.7554/eLife.100396)
Supplement: Supplementary file 4. [file elife-100396-supp4.docx]

**Supplementary File 4. Software and Algorithms**

| FlowJo (v10.0.7) | Flowjo, LLC | https://www.flowjo.com/ |
| --- | --- | --- |
| GraphPad Prism 6 | GraphPad Software Inc. | <https://www.graphpad.com/scien-tific>/software/prism/ |
| ZEN | Carl Zeiss | https://www.zeiss.com/ |
| SCA® CASA System (for Sperm analysis) | MICROPTIC | https://www.micropticsl.com/products/sperm-class-analyzer-casa-system/ |
| Cell Ranger (v3.0.2) | 10x Genomics | https://support.10xgenomics.com/singlecell-gene-expression/software/pipelines/latest/what-is-cell-ranger |
| R software (v4.0.3) | CRAN (Open Source) | https://cran.r-project.org/ |
| Seurat (v4.0) | Satija Lab | https://satijalab.org/seurat/ |
| Monocle (v2.20.0) | Trapnell et al., 2014 | <http://cole-trapnell-lab.github.io/>  monocle-release/ |
| Cellchat | [Suoqin Jin](https://www.nature.com/articles/s41467-021-21246-9#auth-Suoqin-Jin) et al., 2021 | https://github.com/sqjin/CellChat |
| ClusterProfiler (v4.0.5) | Yu G, et al., 2012 | https://bioconductor.org/packages/release/bioc/html/clusterProfiler.html |
| ggplot2 (v3.3.5) | Wickham, et al., 2009 | https://ggplot2.tidyverse.org/ |
| ROSE | Richard A. Young, et al., 2013 | https://github.com/stjude/ROSE |
| MetaboAnalyst 5.0 | Zhou G, et al., 2021 | https://www.metaboanalyst.ca/MetaboAnalyst/ModuleView.xhtml |
