# Supplementary material for: A single-cell atlas of the testicular interstitium defines Leydig progenitor networks sustaining Leydig cell homeostasis across the lifespan
Source: eLife. 2025 Dec 23;14:e100396. doi: 10.7554/eLife.100396 (PMC12826670; doi:10.7554/eLife.100396)
Supplement: Supplementary file 5. [file elife-100396-supp5.docx]

**Supplementary File 5. Critical Commercial Assays**

| CellTrace™ CFSE Cell Proliferation Kit | Thermo Fisher Scientific | Cat# C34554 |
| --- | --- | --- |
| CLICK-IT EDU IMAGING KIT | Thermo Fisher Scientific | Cat# C10086 |
| Testosterone ELISA Kit | Beyotime, China | Cat# PT872 |
