# Supplementary material for: A single-cell atlas of the testicular interstitium defines Leydig progenitor networks sustaining Leydig cell homeostasis across the lifespan
Source: eLife. 2025 Dec 23;14:e100396. doi: 10.7554/eLife.100396 (PMC12826670; doi:10.7554/eLife.100396)
Supplement: Supplementary file 6. [file elife-100396-supp6.docx]

**Supplementary File 6. Instrument and Equipments**

| FACSAria III | BD Bioscience | Country |
| --- | --- | --- |
| Zeiss LSM 780 Confocal Laser Scanning Microscope | Carl Zeiss | Germany |
| Zeiss LSM 900 Confocal Laser Scanning Microscope | Carl Zeiss | Germany |
| Synergy2 | Biotek | USA |
